# *Sophora moorcroftiana* Seeds Ethanol Extract Against Metabolic Dysfunction-Associated Steatotic Liver Disease in Mice by Modulating Gut Microbiota Dysbiosis, SCFAs, and Related Inflammation

**DOI:** 10.3390/ijms27167368

**Published:** 2026-08-18

**Authors:** Xiaotong Chu, Shuang Zhang, Mingxue Cui, Xiaojing Sun, Xiao Chen, Liying Gao, Ruiying Yuan, Sicen Wang, Shan Huang, Bin Li

**Affiliations:** 1 Department of Pharmaceutical Engineering and Pharmacy, School of Chemical Engineering, Qingdao University of Science and Technology, Qingdao 266042, China; 17205228560@163.com (X.C.); zhangshuang9797@163.com (S.Z.); cuimx726@163.com (M.C.); s10240513@163.com (X.S.); 19819197685@163.com (X.C.); ggaoliying@163.com (L.G.); 2 Department of Pharmacy, College of Medicine, Tibet University, Lhasa 850001, China; 1yuan2yuan@163.com (R.Y.); wangsc@mail.xjtu.edu.cn (S.W.); 3 College of Pharmacy, Xi’an Jiaotong University, Xi’an 710061, China

**Keywords:** *S. moorcroftiana* seeds, metabolic dysfunction-associated steatotic liver disease (MASLD), gut microbiota, short-chain fatty acids, gut-liver axis, inflammation

## Abstract

Despite the traditional application of *Sophora moorcroftiana* (Benth.) Baker seeds for liver disorders, the capacity of its 70% ethanol extract (SMS) to alleviate metabolic dysfunction-associated steatotic liver disease (MASLD) and the underlying gut–liver axis mechanisms remain unclear. In this study, a high-fat diet (HFD)-induced MASLD mouse model was established to investigate the protective effects of SMS and its regulatory role in the interplay among gut microbiota, short-chain fatty acids (SCFAs), and inflammation. Serum, intestinal, and hepatic samples were collected to evaluate inflammatory responses, intestinal barrier integrity, and hepatic lipid metabolism. Gut microbiota composition and SCFA profiles were analyzed using 16S rRNA sequencing and metabolomics. In LPS-stimulated Caco-2 cells, SMS reduced inflammatory cytokines and TLR4/MyD88/NF-κB-associated signaling. The results demonstrated that SMS markedly alleviated hepatic steatosis by reducing triglyceride synthesis and hepatocellular lipid accumulation. In addition, SMS promoted the proliferation of beneficial bacteria, including Bifidobacterium and Akkermansia, and increased the production of SCFAs, particularly butyrate. SMS also restored intestinal barrier integrity through upregulation of Occludin and Claudin-1, thereby reducing circulating lipopolysaccharide (LPS) levels. Furthermore, SMS attenuated inflammation by inhibiting activation of the TLR4/NF-κB signaling pathway. Collectively, these findings demonstrate that SMS alleviates MASLD through coordinated modulation of gut microbiota composition, SCFA metabolism, intestinal barrier function, and inflammatory responses, highlighting its potential as a therapeutic strategy targeting the gut–liver axis.

## 1. Introduction

Metabolic dysfunction-associated steatotic liver disease (MASLD), previously known as non-alcoholic fatty liver disease (NAFLD) [1], is defined by the core pathological features of lipid overload within liver cells and the subsequent inflammatory response [2]. The persistent inflammatory state can result in metabolic dysfunction-associated steatohepatitis (MASH), which may further evolve into liver fibrosis, cirrhosis, and eventually hepatocellular carcinoma (HCC) [3]. MASLD pathogenesis is multifactorial, involving dietary habits (particularly high-fat diet), metabolic dysfunction, and genetic susceptibility [4]. Critically, the disruptions in intestinal dysbiosis triggered by a high-fat diet (HFD) are a primary driver of MASLD aggravation [5]. MASLD patients frequently exhibit intestinal flora imbalance, which is characterized by a higher prevalence of Gram-negative bacteria (e.g., *Escherichia coli*), alongside a decrease in beneficial genera like *Coprococcus* [6,7], concomitant with reduced fecal levels of short-chain fatty acids (SCFAs) such as butyrate [8]. Therefore, supplementation with SCFAs has been demonstrated to regulate lipid metabolic disorders and improve liver function in these patients [9]. In addition, the gut microbiota dysbiosis also causes elevation of intestinal lipopolysaccharide (LPS), as the primary ligand for Toll-like receptor 4 (TLR4). It activates TLR4 signaling to promote the secretion of inflammatory mediators, which can reduce the levels of intestinal tight junction proteins, leading to increased epithelial barrier permeability [10]. Consequently, centered on the key bidirectional hub of the “gut–liver axis,” the impaired intestinal barrier permits substantial LPS to translocate to the liver, where it activates TLR4 signaling, triggering a persistent inflammatory response that drives the progression of MASLD. This conclusion is supported by clinical observations of elevated serum LPS levels accompanied by intestinal barrier dysfunction [11,12]. Therefore, restoring microbial homeostasis and enhancing barrier function represents a viable therapeutic approach for alleviating hepatic inflammation and improving liver function in MASLD.

The poly-pharmacology of Traditional Chinese medicine (TCM) offers a distinct advantage for addressing the multifaceted pathology of MASLD [13]. *Sophora moorcroftiana* (Benth.) Baker is a medicinal leguminous plant native to the Qinghai–Tibet Plateau, the Tibetan medical classic “Four Medical Classics” records of *S. moorcroftiana*. In traditional Tibetan medicine, its seeds are recognized for their detoxifying and hepatoprotective properties and have long been used to treat various diseases, including parasitic infections, jaundice hepatitis, and diphtheria. Research has shown that its seeds are a novel potential agent for treating parasitic infections [14]. Our previous research established that SMS relieves CCl_4_-induced liver injury in mice [15]. More recently, we discovered that SMS inhibits hepatic inflammation in MASLD mice by suppressing the ROS-mediated NLRP3 inflammasome activation and pyroptosis [16]. Furthermore, our previous study demonstrated that SMS counteracts MASLD by modulating the AMPK signaling pathway [17].

This study attempts to elucidate the effects of SMS on the gut microbiota composition, SCFA levels, and associated inflammatory responses in HFD-induced mice, thereby clarifying its potential mechanism in alleviating MASLD via the gut–liver axis.

## 2. Results

### 2.1. HPLC Identification and Quantification of Four Alkaloids in SMS

HPLC was used to obtain compositional information on SMS. The four alkaloids were selected because they had been identified in our previous phytochemical studies of SMS and represent matrine-type alkaloids with reported pharmacological relevance. Chromatograms of the four authentic reference standards and SMS are shown in Figure S1A and S1B, respectively, and the corresponding chemical structures are shown in Figure S1C. Comparison with the reference standards and calibration equations identified oxysophocarpine, sophocarpine, matrine, and oxymatrine at 3.99, 0.83, 3.63, and 32.02 mg/g, respectively (Table 1).

**Table 1:** Preliminary HPLC identification and quantification of four alkaloids in SMS.

Compound Name	Molecular Formula	R.T. (min)	Curve Regression Equations	R^2^	Linear Range (μg/mL)	Content (mg/g)
oxysophocarpine	C_15_H_22_N_2_O_2_	16.79	y = 993,723x + 6466	0.9999	45.6~730.0	3.99
sophocarpine	C_15_H_22_N_2_O	13.03	y = 1,489,570.21x + 5504.125	0.9999	21.3~340.0	0.83
matrine	C_15_H_24_N_2_O	9.13	y = 899,472x + 10,421	0.9998	63.9~1022.0	3.63
oxymatrine	C_15_H_24_N_2_O_2_	17.72	y = 344,416x + 8584.3	0.9998	156.3~2500.0	32.02

### 2.2. Effect of SMS on Hepatic Injury, Lipid Deposition, and Inflammation in HFD-Induced MASLD Mice

**Figure 1:**
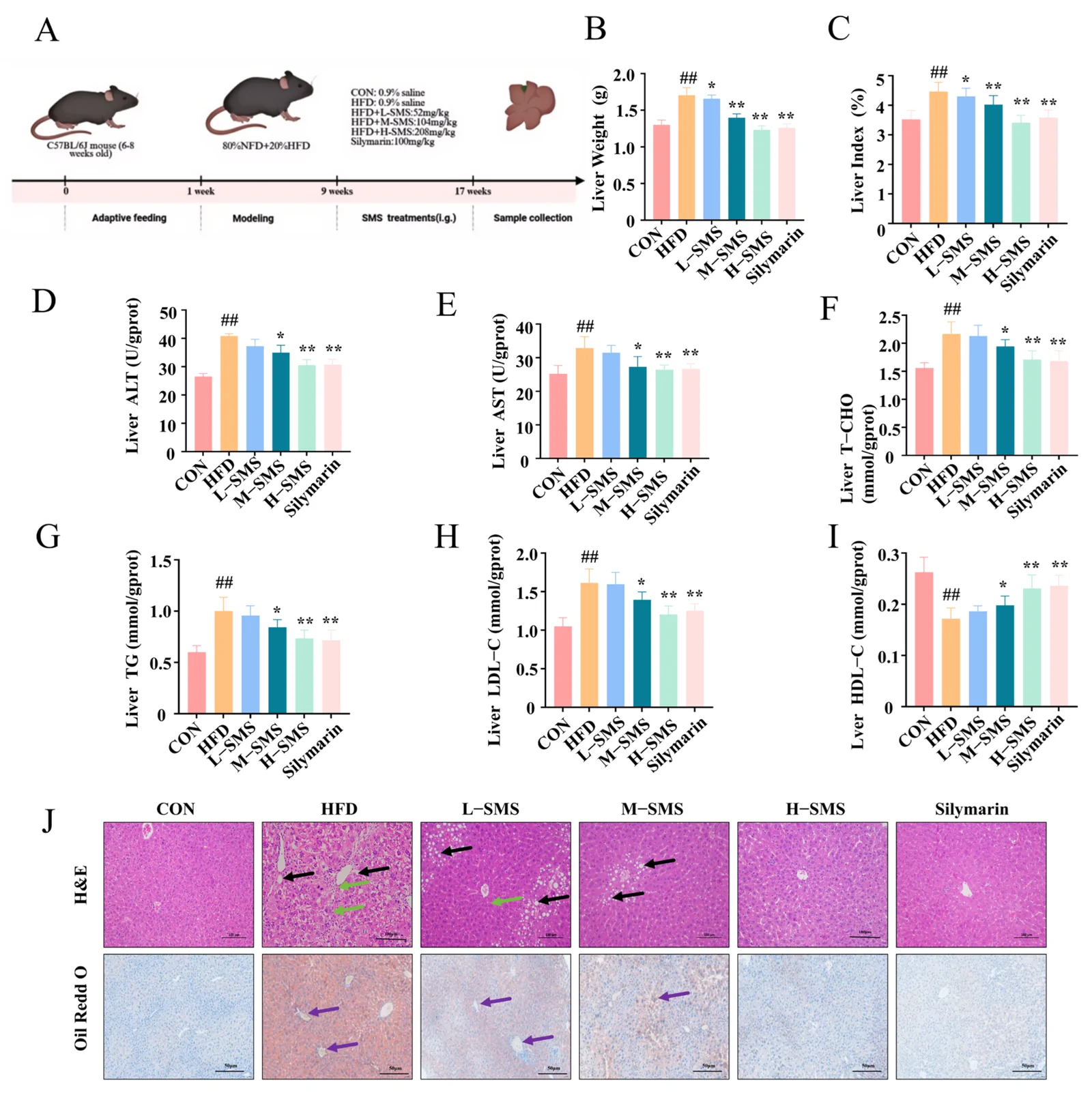
Effect of SMS on hepatic injury and lipid deposition in HFD-induced MASLD mice. (**A**) Animal experimental design. (**B**) Liver weight. (**C**) Liver index, calculated as liver weight/final body weight × 100%. Hepatic (**D**) ALT and (**E**) AST. Measurement of (**F**) T-CHO, (**G**) TG, (**H**) LDL-C, and (**I**) HDL-C in liver tissue. (**J**) H&E staining and Oil Red O staining were performed on liver tissue, (20×). Scale bar = 100 µm. Data are presented as mean ± SD (n = 8). ## *p* < 0.01 compared to the CON group, * *p* < 0.05, ** *p* < 0.01 compared to the HFD group.

All mouse groups received intragastric administration for 8 weeks (Figure 1A). As opposed to the CON (normal-diet control group) group, the HFD group showed elevated liver weight and liver index; these parameters were notably reduced by SMS or Silymarin intervention, particularly in the H-SMS (high-dose SMS group) group (Figure 1B,C). The HFD group showed elevated hepatic alanine aminotransferase (ALT) and aspartate aminotransferase (AST) activities together with histopathological liver injury compared with the CON group, and SMS treatment lowered ALT and AST activities (Figure 1D,E). Furthermore, SMS and Silymarin administration notably diminished the levels of T-CHO (total cholesterol), TG (triglycerides), and LDL-C (low-density lipoprotein cholesterol), while increasing HDL-C levels relative to the HFD group (Figure 1F–I, *p* < 0.05). During treatment, no treatment-related mortality, abnormal behavior, overt clinical signs, or significant treatment-related decrease in body weight was observed. The SMS doses were selected from our previous study using the same extract and HFD model [16]. Overall, SMS treatment improved liver and colon histopathology without aggravating HFD-associated lesions.

As shown in Figure 1J, hepatocytes in the CON group were well-organized, radiating normally from the central vein. In contrast, the HFD group displayed a disrupted hepatic architecture with increased macrovesicular steatosis. Both SMS and Silymarin treatments markedly alleviated these pathological alterations, including hepatocyte steatosis (black arrows), disordered hepatocyte arrangement, and inflammatory cell infiltration (green arrows). Oil Red O staining revealed numerous lipid droplets in the HFD group (purple arrows), whereas SMS treatment substantially reduced this ectopic lipid deposition.

**Figure 2:**
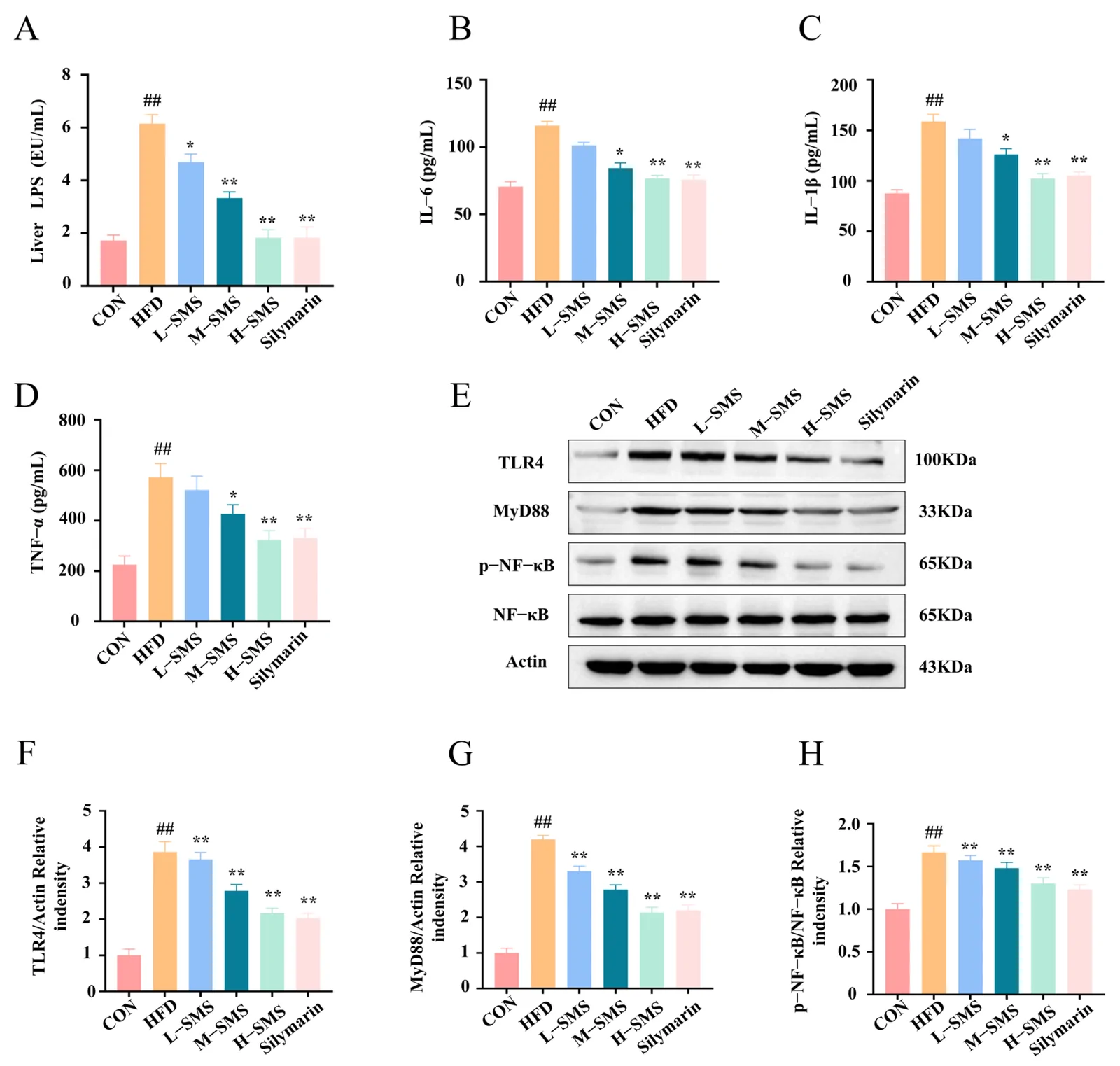
Effect of SMS against hepatic inflammation in HFD-induced MASLD mice. (**A**) LPS concentrations in the liver tissue. The concentration of (**B**) IL-6, (**C**) IL-1β, and (**D**) TNF-α in the liver tissue. (**E**–**H**) Western blot analysis on expressions of TLR4, MyD88, p-NF-κB, and NF-κB protein in the liver tissue. Each lane represents liver tissue lysate from one individual mouse; no samples were pooled. Densitometric values for TLR4, MyD88, and total NF-κB were normalized to β-actin, whereas p-NF-κB was normalized to total NF-κB. Quantification used three biologically independent mice per group (n = 3). Data are presented as mean ± SD (n = 8). ## *p* < 0.01 compared to the CON group, * *p* < 0.05, ** *p* < 0.01 compared to the HFD group.

Consistent with the MASLD phenotype, HFD induced significant hepatic inflammation, as evidenced by heightened concentrations of inflammatory cytokines (IL-1β, IL-6, and TNF-α) (Figure 2B–D) and increased hepatic LPS concentrations (Figure 2A). These inflammatory markers were attenuated by treatment with either SMS or Silymarin. Furthermore, the expression levels of TLR4 pathway proteins were markedly upregulated in the livers of MASLD mice (Figure 2E–H). However, the activation of this pathway was effectively suppressed in both the H-SMS and Silymarin treatment groups. Collectively, these results demonstrate that SMS and Silymarin treatments attenuate hepatic lipid accumulation, reduce inflammation, and confer protection against HFD-induced liver injury in MASLD mice.

### 2.3. Effect of SMS on the Colon Morphology and Intestinal Permeability of the HFD-Induced MASLD Mice

**Figure 3:**
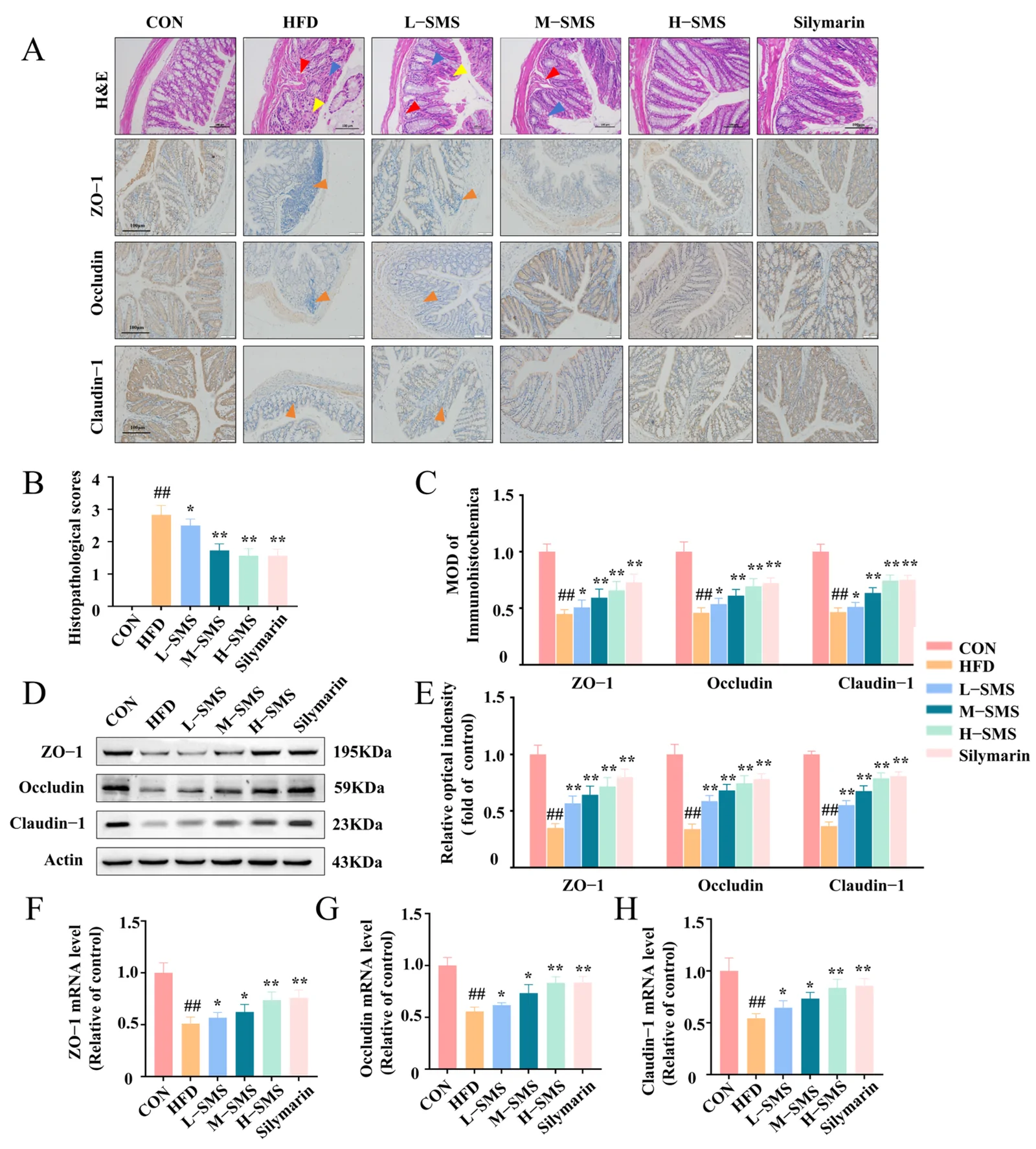
Effect of SMS on the colon morphology and tight junction of the colon in HFD-induced MASLD mice. (**A**–**C**) Colon H&E staining and IHC staining of tight junction proteins (ZO-1, Occludin and Claudin-1) (20×). Scale bar = 100 µm, with corresponding quantitative analysis (red arrows indicate destruction of the intestinal mucosa; blue arrows indicate inflammatory infiltration; yellow arrows indicate the disappearance of goblet cells and swelling and rupture of the crypts; orange arrows indicate the loss of corresponding tight junction protein expression). (**D**,**E**) Western blot analysis on expressions of ZO-1, Occludin, and Claudin-1 protein in the colon tissue. Each lane represents colon tissue lysate from one individual mouse; no samples were pooled. Densitometric values were normalized to β-actin, and quantification used three biologically independent mice per group (n = 3). (**F**–**H**) Relative expression levels of colon tissue ZO-1, Occludin, and Claudin-1 mRNA. Data are presented as mean ± SD (n = 8). ## *p* < 0.01 compared to the CON group, * *p* < 0.05, ** *p* < 0.01 compared to the HFD group.

Colon tissue by H&E staining (Figure 3A,B and Figure S2A–D) demonstrated that a HFD induced characteristic pathological alterations, such as inflammatory cell infiltration (yellow arrows), crypt architectural distortion, depletion of goblet cells (blue arrows), and mucosal injury (red arrows). These HFD-induced pathological changes were markedly alleviated by SMS treatment. Concordant with the histological improvements, SMS administration reduced the levels of permeability biomarkers in colon tissue (Figure 3D–H and Figure S2E–H). IHC analysis revealed that HFD feeding substantially downregulated the expression of key tight junction proteins (orange arrows), while SMS intervention effectively restored their expression levels (Figure 3A–C). This regulatory effect of SMS was further corroborated by Western blot and qRT-PCR analyses. Based on these collective findings, SMS exerts a restorative effect on the intestinal barrier in MASLD mice by upregulating the expression of intestinal tight junction proteins.

### 2.4. Effect of SMS on the Inflammation, Barrier Structure and Integrity of the Colon in HFD-Induced MASLD Mice

**Figure 4:**
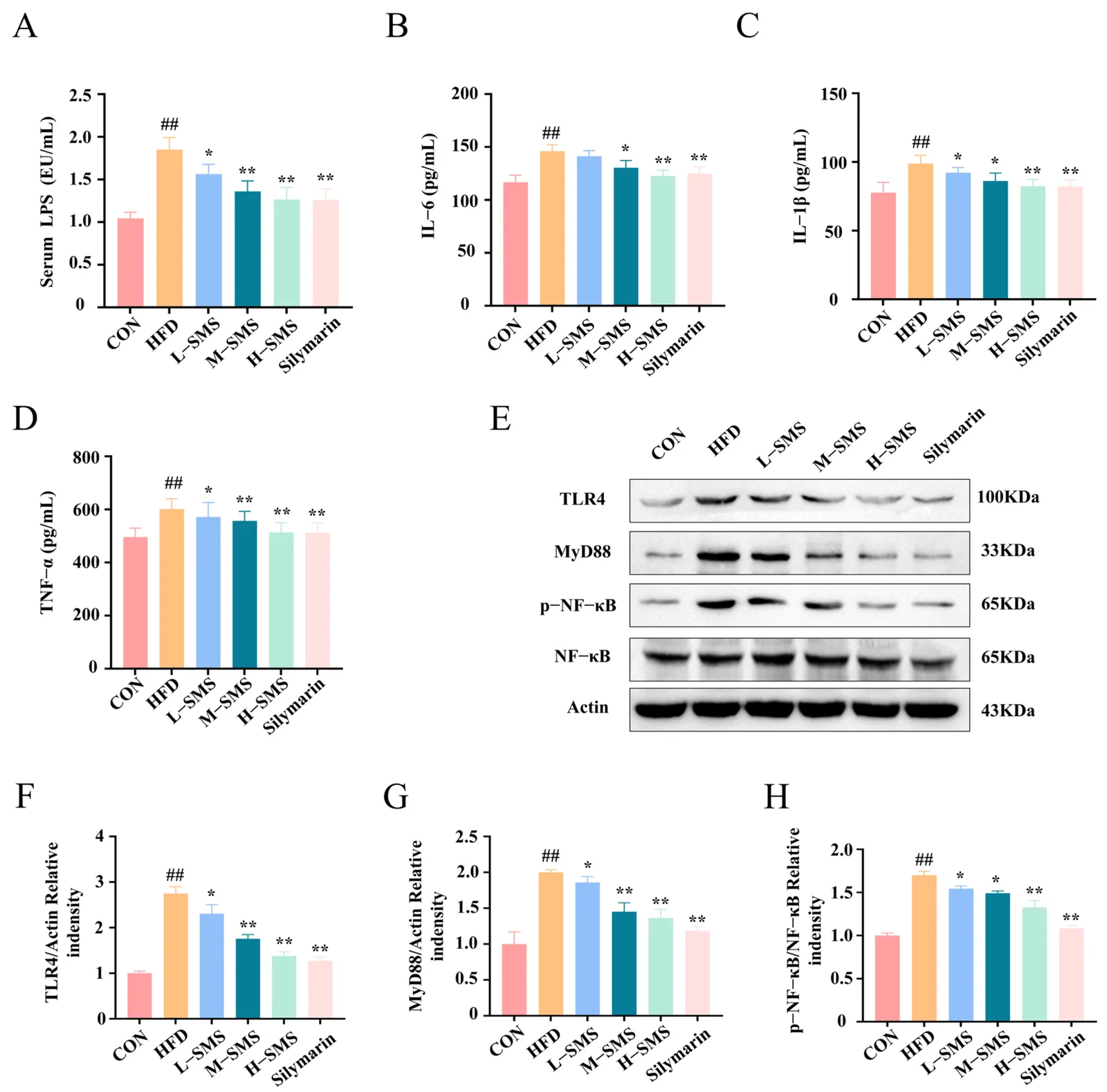
Effect of SMS on TLR4/NF-κB-mediated inflammation in HFD-induced MASLD mice. (**A**) LPS concentrations in serum. The concentration of (**B**) IL-6, (**C**) IL-1β, and (**D**) TNF-α in the colon tissue. (**E**–**H**) Western blot analysis on expressions of TLR4, MyD88, and p-NF-κB/NF-κB protein in the colon tissue. Each lane represents colon tissue lysate from one individual mouse; no samples were pooled. Densitometric values for TLR4, MyD88, and total NF-κB were normalized to β-actin, whereas p-NF-κB was normalized to total NF-κB. Quantification used three biologically independent mice per group (n = 3). Data are presented as mean ± SD (n = 8). ## *p* < 0.01 compared to the CON group, * *p* < 0.05, ** *p* < 0.01 compared to the HFD group.

**Figure 5:**
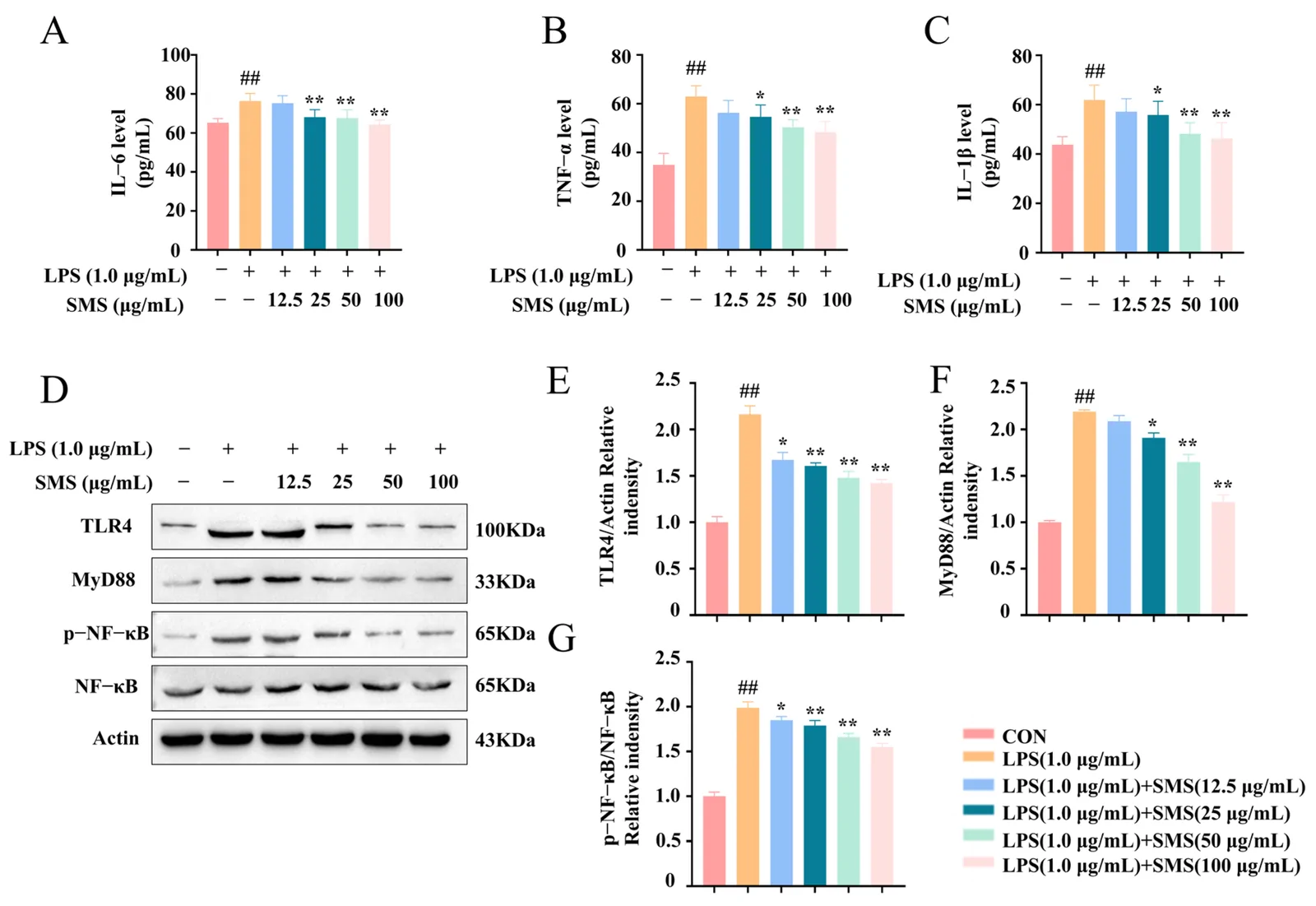
Impact of SMS on TLR4/NF-κB-mediated restorative effects of the intestinal barrier in LPS-induced Caco-2 cells. (**A**–**C**) The concentration of IL-6, TNF-α, and IL-1β in LPS-induced Caco-2 cells treated with SMS. (**D**) Representative Western blots of TLR4, MyD88, p-NF-κB, NF-κB, and β-actin. (**E**–**G**) Densitometric quantification of TLR4/β-actin, MyD88/β-actin, and p-NF-κB/NF-κB. Data are mean ± SD from three independent experiments (n = 3); three technical replicate wells per treatment condition were averaged within each experiment. ## *p* < 0.01 compared to the CON group, * *p* < 0.05, ** *p* < 0.01 compared to the HFD group.

Increased intestinal permeability due to impaired gut barrier function facilitates the translocation of LPS. Considering the gut microbiota serves as a primary source of LPS, we hypothesized that SMS suppresses LPS production by modulating the gut microbiota in MASLD mice. Administration of a HFD markedly elevated systemic LPS levels in mice compared to the CON group, an effect that was effectively reversed by SMS treatment (Figure 4A). Concurrently, SMS intervention substantially reduced the concentration of pro-inflammatory cytokines in the colon (Figure 4B–D). Western blot analysis further demonstrated that SMS downregulated the expression of pathway-associated proteins in colon tissue (Figure 4E–H). Consistent with these in vivo observations, in vitro, co-treatment with 25 μg/mL SMS significantly attenuated the inflammatory response (Figure 5A–C) and the upregulation of the above pathway proteins induced by LPS stimulation (Figure 5D–G). Collectively, these findings indicate that SMS suppresses inflammation in MASLD likely by reducing systemic LPS translocation and subsequently inhibiting the activation of the signaling cascade.

### 2.5. SMS Attenuates LPS-Induced Inflammatory Responses in Caco-2 Cells

To complement the in vivo inflammatory findings, we used LPS-stimulated Caco-2 cells. Exposure to 1.0 μg/mL LPS reduced cell viability, whereas SMS concentrations up to 100 μg/mL did not significantly affect viability under the tested conditions (Figure S3A,B). Accordingly, SMS concentrations of 12.5, 25, 50, and 100 μg/mL were evaluated. SMS lowered IL-6, TNF-α, and IL-1β concentrations and reduced TLR4 and MyD88 abundance and NF-κB phosphorylation relative to LPS alone (Figure 5A–G). These results show that SMS attenuated the measured LPS-induced inflammatory responses in Caco-2 cells.

### 2.6. Effect of SMS on Gut Microbiota Diversity, Composition and Change in Abundance in HFD-Induced MASLD Mice

**Figure 6:**
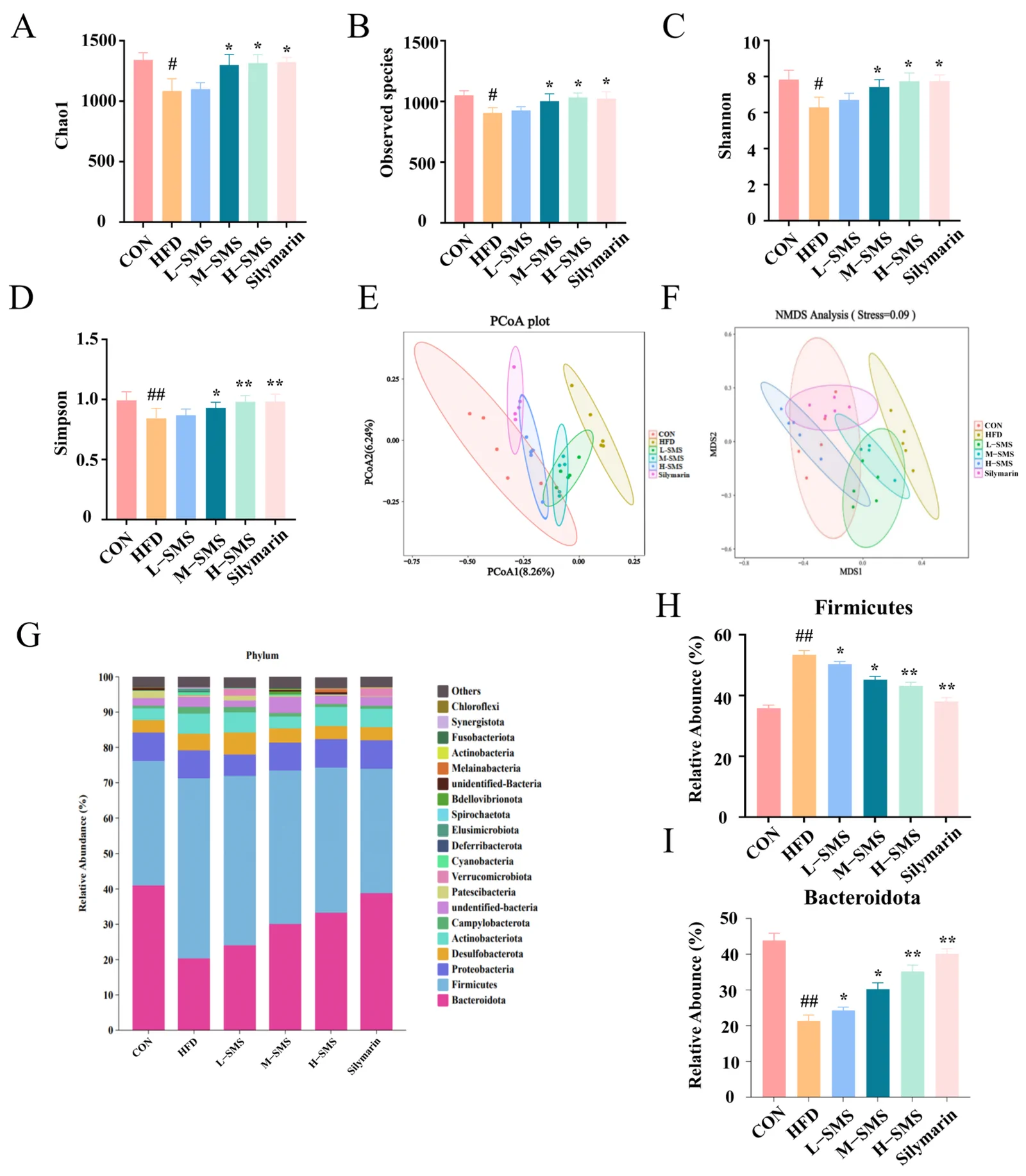
Effect of SMS on gut microbiota composition and diversity in HFD-induced MASLD mice. Analysis of (**A**) Chao1, (**B**) observed species, (**C**) Shannon and (**D**) Simpson indices for alpha diversity in each group. (**E**) PCoA and (**F**) NMDS analysis of beta diversity. (**G**) Relative abundance of the top 20 representative microorganisms at the phylum level. Relative histogram of (**H**) Firmicutes and (**I**) Bacteroidota at phylum level. Data are presented as mean ± SD (n = 5). # *p* < 0.05, ## *p* < 0.01 compared to the CON group, * *p* < 0.05, ** *p* < 0.01 compared to the HFD group.

**Figure 7:**
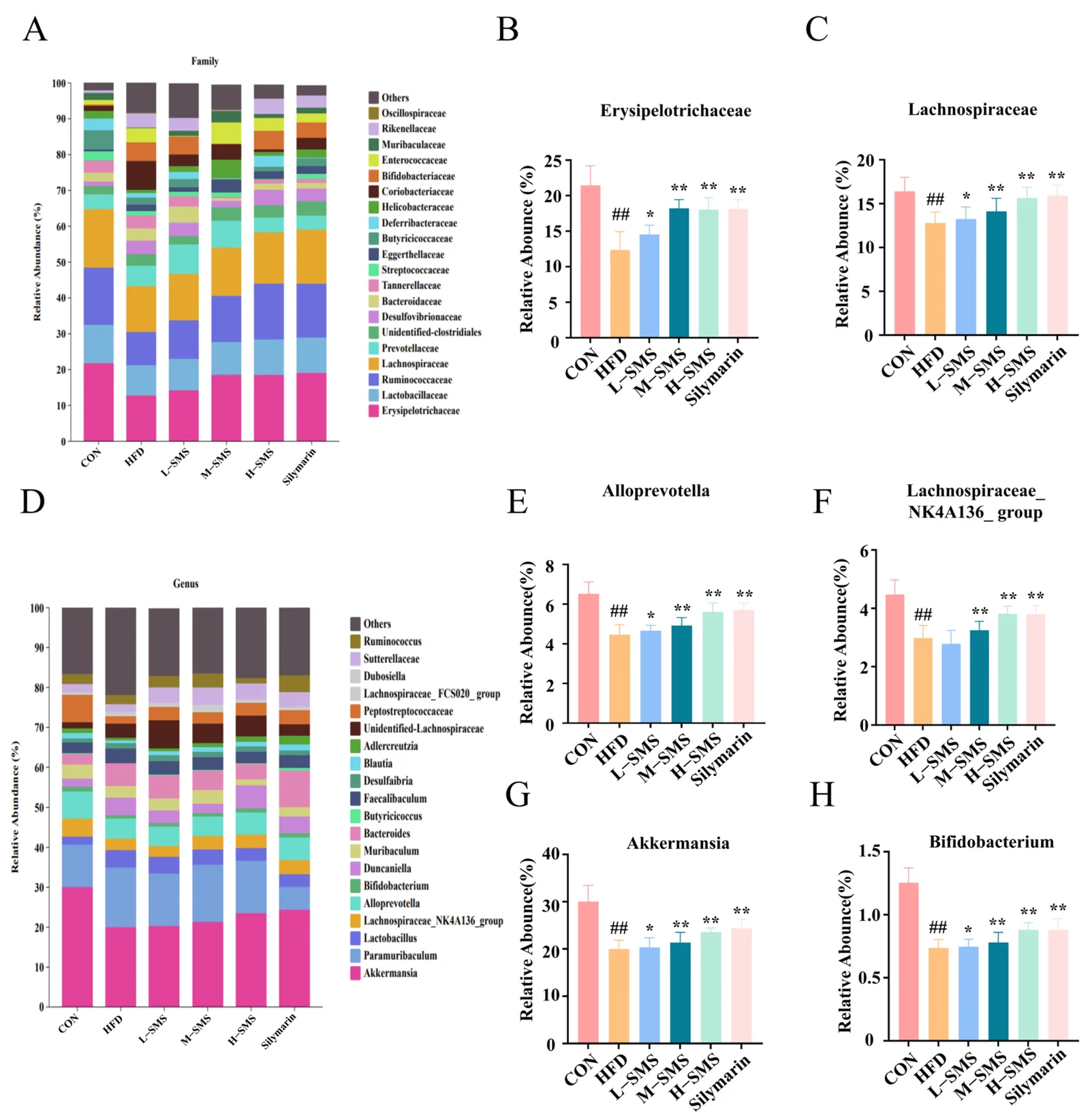
Effect of SMS on intestinal microbiota change in abundance at the family and genus levels in HFD-induced MASLD mice. (**A**) Relative abundance of the top 20 representative microorganisms at the family level. Relative histogram of (**B**) Erysipelotrichaceae and (**C**) Lachnospiraceae at the family level. (**D**) Relative abundance of the top 20 representative microorganisms at the genus level. Relative histogram of the (**E**) *Alloprevotella*, (**F**) Lachnospiraceae_NK4A136_group, (**G**) *Akkermansia*, and (**H**) *Bifidobacterium* at the genus level. Data are presented as mean ± SD (n = 5). ## *p* < 0.01 compared to the CON group, * *p* < 0.05, ** *p* < 0.01 compared to the HFD group.

SMS treatment enhanced microbial alpha diversity, as evidenced by increased Chao1, observed species, Simpson, and Shannon indices (Figure 6A–D). Beta-diversity analysis via Principal Coordinate Analysis (PCoA) and Non-Metric multidimensional scaling (NMDS) revealed distinct clustering patterns among the experimental groups (Figure 6E,F), indicating structural separation of the gut microbial communities. At the phylum level, Firmicutes and Bacteroidetes were the most abundant taxa (Figure 6G–I). Furthermore, taxonomic analysis at the genus and family levels demonstrated that SMS supplementation enriched beneficial bacteria, including *Alloprevotella, Akkermansia*, *Bifidobacterium*, and *Lachnospiraceae*_NK4A136_group, while reversing HFD-induced declines in *Erysipelotrichaceae* and *Lachnospiraceae* (Figure 7). Altogether, these findings indicate that SMS restores gut microbial diversity and favorably modulates community structure in MASLD mice.

### 2.7. Effects of SMS on LEfSe Analysis and Metabolic Functions of Gut Microbiota

**Figure 8:**
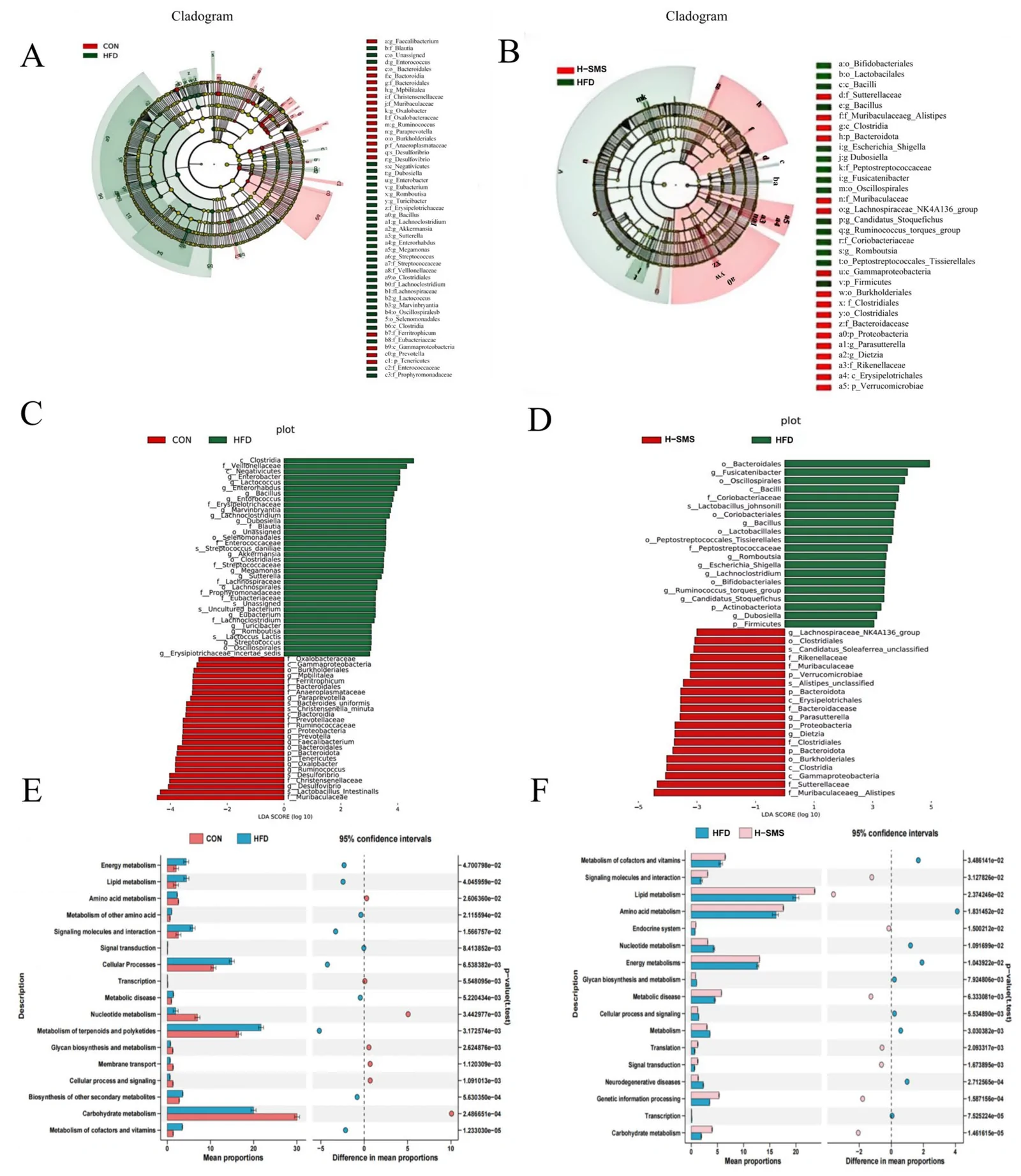
Effect of SMS on linear discriminant analysis effect size (LEfSe) analysis and metabolic functions of gut microbiota. (**A**,**B**) LEfSe analysis and (**C**,**D**) LDA score for characteristic bacterial taxa. (**E**,**F**) Significant functional abundance differences between CON, HFD and H-SMS in KEGG pathway. The CON and HFD groups (**A**,**C**,**E**). The HFD and H-SMS groups (**B**,**D**,**F**).

The linear discriminant analysis effect size (LEfSe) method (LDA score > 3) revealed distinct microbial taxa that significantly differed among the experimental groups (Figure 8A–D and Figure S4). The gut microbial profile of the HFD group was marked by a higher abundance of Proteobacteria and Firmicutes, which was largely attributable to an increase in the families f_Desulfovibrionaceae and f_Lachnospiraceae. Conversely, this group exhibited a decline in the abundance of f_Bacteroidales_S24-7_group (Bacteroidetes) and f_Ruminococcaceae (Firmicutes). Silymarin supplementation led to an enrichment of g_*Bacteroides*, f_Lachnospiraceae, and g_Lachnospiraceae_NK4A136_group. Meanwhile, the H-SMS group showed a pronounced increase in f_Erysipelotrichaceae, p_Bacteroidota, g_*Bacteroides*, and f_Lachnospiraceae. These modifications reflected a considerable restructuring of the gut microbial composition relative to the HFD group.

Phylogenetic Investigation of Communities by Reconstruction (PICRUSt) analysis assessed functional variations in the gut microbiota by examining the top 17 differential Kyoto encyclopedia of genes and genomes (KEGG) pathways at level 2. As shown in Figure 8E, HFD mice exhibited significant alterations in multiple functional categories, including lipid metabolism, carbohydrate metabolism, signaling, membrane transport, endocrine system, and amino acid synthesis and metabolism pathways. Intervention with Silymarin notably enhanced functions related to transcription, membrane transport, and cellular signaling pathways (Figure S4C). In contrast, SMS treatment primarily enriched metabolic pathways, including carbohydrate metabolism, fatty acid regulation, and the biosynthesis of signaling molecules and amino acids (Figure 8F).

### 2.8. Effect of SMS on SCFA Contents in HFD-Induced MASLD Mice

**Figure 9:**
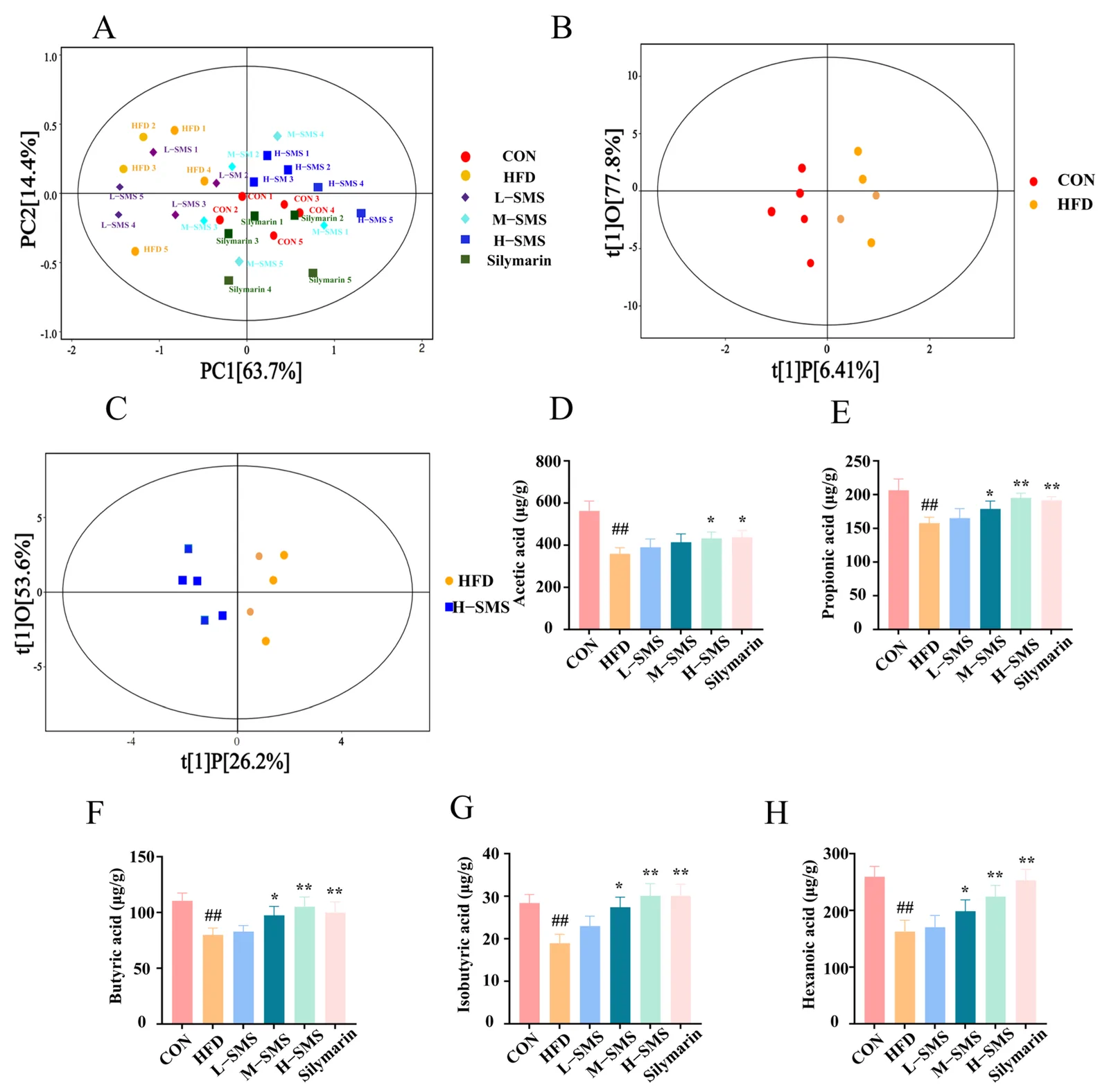
Effect of SMS on the metabolic profile of SCFAs in HFD-induced MASLD mice. (**A**) The PCA score plot in each group. (**B**–**C**) The OPLS-DA score plot of the CON vs. HFD groups and the HFD vs. H-SMS groups. (**D**–**H**) Changes in SCFA content in feces of mice in each group, including acetic, propionic, butyric, isobutyric and hexanoic acid. Data are presented as mean ± SD (n = 5). ## *p* < 0.01 compared to the CON group. * *p* < 0.05, ** *p* < 0.01 compared to the HFD group.

Principal component analysis (PCA) revealed a clear separation among the experimental groups, indicating distinct profiles of SCFA in each group (Figure 9A and Figure S5). Consistent with this, orthogonal partial least squares discriminant analysis (OPLS-DA) demonstrated distinct clustering of SCFA components among the CON, HFD, and various SMS-treated groups (Figure 9B,C and Figure S5A–C). Notably, the SMS-treated groups showed a compositional shift towards the CON group in the score plots. Subsequent quantitative analysis of SCFA levels showed that, compared to the CON group, HFD-fed mice had significantly decreased concentrations of SCFAs in colonic contents (Figure 9D–H). Importantly, SMS treatment dose-dependently reversed this reduction and elevated SCFA concentrations. In conclusion, these findings suggest that SMS restores SCFA production in MASLD mice, which may contribute to the preservation of intestinal barrier function.

### 2.9. Spearman Correlation Analysis

**Figure 10:**
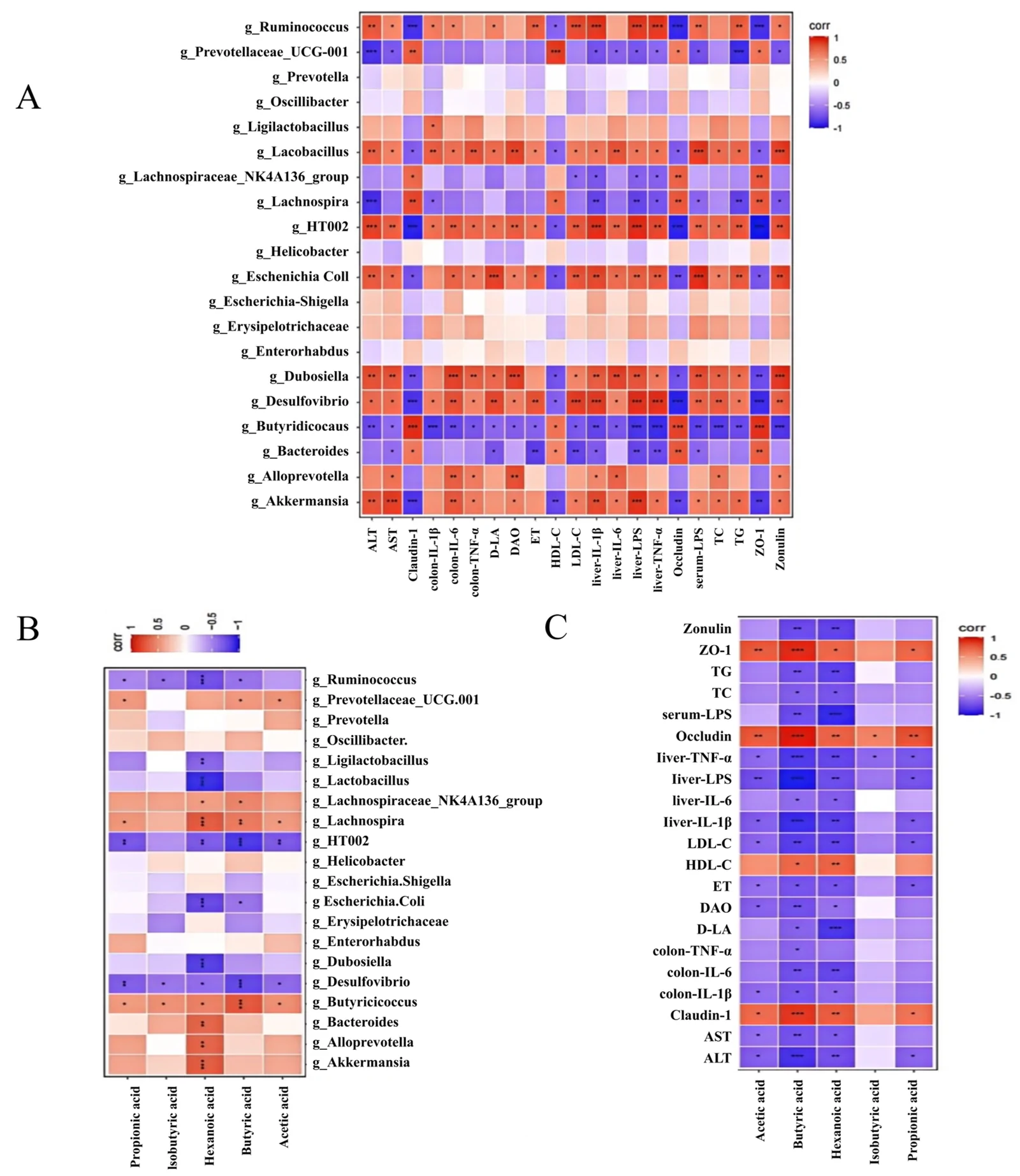
Spearman correlation analysis. (**A**) Correlation heatmap for the relative abundances of gut microbiota at genus level and core parameters. (**B**) Correlation heatmap for the relative abundance of gut microbiota at the genus level and the content of SCFAs. (**C**) Correlation heatmap for the content of SCFAs and the core parameters. The area marked by an asterisk indicates * *p* < 0.05, ** *p* < 0.01 and *** *p* < 0.001.

Spearman correlation analysis revealed significant associations between key gut bacteria and clinical parameters in MASLD (Figure 10A). *Alloprevotella*, *Lactobacillus*, and *Bifidobacterium* were inversely correlated with hepatic lipids and pro-inflammatory cytokines (*p* < 0.05). Conversely, *Rikenella*, Lachnospiraceae_NK4A136_group, and Lachnospiraceae_UCG-008 showed positive correlations with these parameters. Specifically, *Lactobacillus* abundance was negatively linked to AST levels (*p* < 0.05), while Lachnospiraceae_UCG-008 was positively associated with ALT (*p* < 0.05). Furthermore, *Desulfovibrio* and *E. coli* exhibited a positive association with systemic and hepatic LPS levels (*p* < 0.01). Additionally, Ruminococcaceae abundance was positively correlated with intestinal permeability markers (ET, DAO) but negatively linked to the tight junction protein ZO-1.

Spearman correlation analysis was performed to evaluate the associations between specific bacterial taxa and changes in SCFA metabolism (Figure 10B). Lachnospiraceae_NK4A136_group, *Bacteroides*, *Akkermansia*, *Alloprevotella*, and *Butyricicoccus* showed positive correlations with acetic, butyric, and hexanoic acid levels. In contrast, *Ligilactobacillus*, *Dubosiella*, and *Desulfovibrio* were negatively correlated with acetic, propionic, and butyric acid concentrations (*p* < 0.05). Additionally, the abundances of *Bacteroides* were positively associated with isobutyric acid levels (*p* < 0.05).

Analysis of SCFA levels revealed significant correlations with key therapeutic indices (Figure 10C). Acetate, propionate, and butyrate positively correlated with tight junction protein expression, whereas butyrate alone was negatively correlated with gut permeability markers. Propionate and butyrate were also associated with improved metabolic and inflammatory profiles, showing negative correlations with liver lipids, injury markers, and pro-inflammatory cytokines, but a positive correlation with HDL-C (*p* < 0.05). A particularly strong negative correlation was observed between butyrate and serum or liver LPS (*p* < 0.01). Collectively, these results indicate that SMS exerts its therapeutic effect against MASLD by modulating the gut microbiota to increase SCFAs, strengthening the intestinal barrier, and mitigating subsequent hepatic inflammation and injury.

## 3. Discussion

This study demonstrates that the ameliorative effect of SMS on MAFLD is achieved through multi-target mechanisms, including enhancing intestinal barrier integrity, modulating specific gut microbiota, restoring SCFA levels, and inhibiting LPS-mediated related inflammatory responses.

The gut–liver axis constitutes a bidirectional relationship among the intestinal tract, resident microbiome, and liver. This interaction helps maintain physiological hepatic activity and delay the advancement of hepatic diseases by modulating the gut microbiota to restore gut–liver homeostasis [18]. Notably, the intestinal microbial community is primarily dominated by the phyla Firmicutes and Bacteroidetes, both of which perform critical functions in upholding intestinal homeostasis [19]. Clinical data have indicated an abnormal enrichment of Firmicutes in adults with MASLD, a finding corroborated in MASLD mice [20,21]. Administration of SMS significantly reduced the abundance of Firmicutes and increased the proportion of Bacteroidetes, contributing to the restoration of gut microbial balance. Furthermore, a decreased abundance of Lactobacillus is a consistent feature in both MASLD patients and mice [22,23]. The Tianhuang formula (TH) alleviates MASLD symptoms in mice by modulating the gut microbiota and increasing the abundance of Lactobacillus [24]. Our findings indicated that SMS had a beneficial effect on the growth of Lactobacillus. Additionally, an increase in the abundance of Alloprevotella has been shown to promote intestinal epithelial cell proliferation and restore the intestinal barrier function in MASLD mice [8]. The improvement in intestinal barrier function by SMS is likely related to the proliferation of Alloprevotella. Another study revealed an enrichment of Desulfovibrio in the gut of MASLD mice, which impaired the intestinal barrier and exacerbated liver inflammation [25]. Our results indicate that SMS inhibits the abundance of Desulfovibrio, correlating with an alleviation of hepatic inflammation. It has been reported that the abundance of *Escherichia coli* is elevated in the gut of MASLD patients compared to healthy individuals, and this observation has been corroborated in MASLD mice [26,27]. LPS, derived from the cell wall of *E. coli*, has the capacity to disrupt tight junction integrity within the gut, leading to increased intestinal permeability. This allows *E. coli* and LPS to translocate to the liver via the portal circulation, activating hepatic inflammatory signaling pathways (e.g., TLR4) and exacerbating the inflammatory response [28]. Our findings demonstrate that the improvement in intestinal and hepatic pathological indicators in MASLD mice by SMS is also linked to the suppression of abnormal *E. coli* proliferation. Consequently, the amelioration of gut microbiota homeostasis by SMS is a key mechanism underlying its therapeutic effect on MASLD.

Instrumental in colonic homeostasis, SCFAs are microbial metabolites that supply energy to the lining of the colon, thereby reinforcing intestinal barrier function and modulating inflammatory responses through their anti-inflammatory properties [29]. Furthermore, SCFAs modulate hepatic lipid metabolism via the gut–liver axis, thus influencing the progression of MASLD [19]. The production of SCFAs is closely associated with bacterial genera such as *Limosilactobacillus fermentum* and Bifidobacterium [30,31]. In MASLD mice, oral administration of Bifidobacterium alleviated hepatic inflammation and lipid accumulation, which was potentially linked to increased intestinal levels of SCFAs, particularly butyrate [32]. Likewise, *Limosilactobacillus fermentum* exhibited a comparable ameliorative effect. Growing evidence indicates that the three primary short-chain fatty acids (such as acetate, propionate, and butyrate) can mitigate inflammation associated with MASLD. Acetate has been shown to reduce liver steatosis and mitigate inflammatory infiltration in MASH mice [33]. Propionate directly inhibits hepatic cholesterol and TG synthesis in MASH mice, therefore regulating systemic lipid metabolism disorders [34]. Butyrate primarily exerts its beneficial effects through its anti-inflammatory activity and its role in repairing the intestinal barrier. It inhibits LPS or endotoxin translocation and reduces the production of pro-inflammatory cytokines, consequently alleviating inflammation in both the gut and the liver [35]. Butyrate also upregulates the expression of colonic tight junction proteins, such as ZO-1, in MASLD mice, effectively enhancing intestinal barrier integrity [36]. Administration of butyrate was confirmed to attenuate liver injury in T2DM-related MASLD mice, an effect associated with the regulation of intestinal epithelial tight junction proteins [37]. In vivo, SMS increased SCFA concentrations in colonic contents, reduced circulating LPS and intestinal inflammation, increased the expression of intestinal tight junction proteins, and decreased zonulin levels, supporting its beneficial effects on intestinal barrier-associated outcomes in HFD-induced MASLD mice. Collectively, these results support the conclusion that SCFAs mitigate the progression of MASLD by regulating liver lipid homeostasis, inhibiting inflammatory activation, and reinforcing the intestinal epithelial barrier.

Chronic inflammation and intestinal barrier dysfunction, driven by gut microbiota dysbiosis, are key factors in the pathogenesis of MASLD [38,39]. These processes are accompanied by abnormal increases in gut-derived LPS [40]. LPS impairs the intestinal epithelial barrier by disrupting tight junction integrity, facilitating its translocation to the liver. Subsequent recognition of LPS by TLR4 triggers a cascade of cytokines, resulting in the production of key inflammatory mediators that drive hepatic inflammation [41]. Our study demonstrates that SMS can ameliorate MASLD by maintaining gut microbiota homeostasis, suppressing LPS generation, and blocking the TLR4/NF-κB-mediated inflammatory cascade, which alleviate inflammation in both the gut and the liver. Further evidence shows that SMS upregulates key tight junction proteins and reduces zonulin levels, hence ameliorating intestinal damage in MASLD mice. Furthermore, previous intervention studies have shown that acetate and butyrate suppress LPS-induced inflammatory responses in Caco-2 cells, including reductions in TNF-α, IL-6, and IL-1β expression [42,43,44]. These findings provide direct evidence for the anti-inflammatory effects of SCFAs on intestinal epithelial cells. These findings collectively indicate that SMS contributes to the restoration of intestinal function and gut homeostasis, decreasing the production of LPS by gut microbes, and subsequently mitigates MASLD progression.

Our previous phytochemical analysis utilizing UPLC-MS identified that the primary active constituents of SMS primarily comprise alkaloids, flavonoids, and other compounds [15,17]. It is noteworthy that alkaloid compounds demonstrate a broad spectrum of pharmacological activities, such as anti-infective and anti-inflammatory effects, as well as the ability to regulate gut dysbiosis. For instance, sophocarpine potentially inhibits cancer-related inflammation through the regulation of intestinal microbial dysbiosis [45]. Oxymatrine contributes to immunomodulation by restoring microbial equilibrium [46], whereas matrine alleviates liver fibrosis via the gut–liver axis in mice [47]. Based on the collective evidence, these findings reveal the material basis by which SMS alleviates MASLD through modulation of the gut microbiota and the gut–liver axis. However, SMS contains other constituents that may also contribute to its biological effects. Further LC–MS profiling and related pharmacological studies will therefore be conducted to characterize these constituents and clarify their respective contributions to the activity of SMS.

## 4. Materials and Methods

### 4.1. Reagents and Materials

ELISA kits for interleukin-6 (IL-6, EK0411), interleukin-1β (IL-1β, EK0394), and tumor necrosis factor-α (TNF-α, EK0527) were sourced from Boster Biological Technology Co., Ltd. (Wuhan, China). Commercial kits of aspartate aminotransferase (AST, C010-2-1), alanine aminotransferase (ALT, C009-2-1), triglyceride (TG, A110-1-1), total cholesterol (T-CHO, A111-1-1), low-density lipoprotein cholesterol (LDL-C, A113-1-1) and high-density lipoprotein cholesterol (HDL-C, A112-1-1) were purchased from Jiancheng Research Institute Co., LTD (Nanjing, China). Reagent test kits for D-lactic acid (D-LA, ml895244), diamine oxidase (DAO, ml300742), endotoxin (ET, ml298875), and zonulin (ml624990) were procured from Shanghai Enzyme-linked Biotechnology Co., Ltd. (Shanghai, China). The LPS (CSB-E13066m) test kit was purchased from Cusabio technology LLC (Houston, TX, USA). RIPA (HY-K1001) lysis buffer was purchased from MedChemExpress (Shanghai, China). Phenylmethylsulfonyl fluoride (PMSF, P0100) and primary antibodies including Occludin (DF7504), Claudin-1 (AF0127), Zonula occludens-1 (ZO-1, AF5145), toll-like receptors-4 (TLR4, AF7017), medullary differentiation factor 88 (MyD88, AF5195), phosphorylated nuclear factor kappa-B (p-NF-κB, AF3219), nuclear factor kappa-B (NF-κB, BF8005), and Actin (240155) were acquired from Zenbiosciences (Chengdu, China). Matrine (≥98% purity, A0094), Oxymatrine (≥98%, A0095), Sophocarpine (≥98%, A0081), and Oxysophocarpine (≥98%, A0520) were obtained from Chengdu Must Bio-Technology Co., Ltd. (Chengdu, China), while the remaining chemicals were sourced from Merck KGaA (Darmstadt, Germany).

### 4.2. Sample Preparation

The herbal material of Sophora moorcroftiana seeds was acquired from Lhasa (Tibet, China) in March of 2022 and authenticated by Professor Zhuoma Dongzhi. The voucher specimens (No. utibet-1539) were deposited at the Herbarium, College of Medicine, Tibet University, Lhasa, China. The powdered *S. moorcroftiana* seeds was extracted twice with 70% ethanol at a 1:10 (*w*/*v*) ratio under reflux for 1 h per extraction. After cooling, the extracted solutions were combined, vacuum-filtered, and concentrated under reduced pressure to obtain the SMS (yield: 20.44%).

### 4.3. High-Performance Liquid Chromatography (HPLC) Analysis

HPLC analysis was performed on an LC-2010A HT HPLC system. The chemical components were separated on a ZORBAX XDB-C18 column (100 × 4.6 mm, 3.5 μm). The reference standards and the SMS sample were separately dissolved in methanol, and then injected into the chromatographic column for separation. The detailed chromatographic conditions for the HPLC analysis of SMS are provided in Table 2.

### 4.4. Animals

Male C57BL/6J mice (20 ± 2 g) were purchased from Pengyue Experimental Animal Breeding Co., Ltd. (Jinan, China; License No. SCXK (Lu) 20220006) and housed under specific conditions at 25 ± 2 °C and 50–60% humidity, with a 12 h light/dark cycle and free access to food and water. All experimental procedures were approved by the Institutional Ethics Committee of Qingdao University of Science and Technology (approval number: QKDLL2024-21).

### 4.5. Animal Models and Drug Administration

After an adaption period of one week, mice were randomized into a normal group (CON group, n = 8) and a model group (n = 40). For eight weeks, the model group mice were fed a 20% HFD (3% cholesterol, 7% lard, and 10% egg yolk powder), while the CON group received a common chow diet.

Subsequently, the model group mice were randomly divided into five groups (n = 8/group): HFD, positive control (Silymarin, 100 mg/kg), L-SMS (low-dose SMS group) (52 mg/kg), M-SMS (medium-dose SMS group) (104 mg/kg) and H-SMS (208 mg/kg). Dosage was converted based on body surface area equivalence between humans and animals [48]. The respective therapeutic agents were administered to each treatment group by oral gavage. The gavage volume was 1 mL/100 g, administered once daily for 8 weeks. SMS doses were selected to match a previous pharmacological study using the same extract and disease model [16]. The 104 mg/kg dose was used as the reference level, with 52 and 208 mg/kg representing 0.5- and 2-fold doses, respectively. After a 12 h fast, samples of colonic contents and fresh feces were collected from five randomly selected mice per group and subjected to 16S rRNA sequencing analysis. Mice were anesthetized with an intraperitoneal injection of 0.3% pentobarbital sodium (50 mg/kg). Serum was separated from orbital blood; the liver was removed and weighed. A section of the left lobe was fixed in 4% paraformaldehyde, with the residual liver, colon, and fecal samples reserved for further examination.

### 4.6. Histopathological Examinations

Liver and colon tissue samples were dehydrated through ethanol, embedded in paraffin, and sectioned at a thickness of 4 μm. The sections were stained with hematoxylin and eosin (H&E). The severity of colonic injury was assessed by histopathological score [49].

### 4.7. Oil Red O Staining

Liver cryosections were immersed in 60% isopropanol and subsequently exposed to an Oil Red O working solution for 15 min in darkness. After differentiation in 60% isopropanol and a deionized water rinse, the sections were counterstained with hematoxylin and washed under running tap water for 10 min, before being examined under a light microscope (Olympus Corporation DP74, Tokyo, Japan).

### 4.8. Immunohistochemical (IHC) Assay

Following deparaffinization and rehydration, sections of paraffin-embedded intestinal tissue underwent antigen retrieval by heating in citrate buffer. After blocking, sections were processed with primary antibody and secondary antibody, followed by chromogenic development and hematoxylin counterstaining. Imaging was conducted using a microscope.

### 4.9. 16S rRNA Gene Sequencing

Total genomic DNA was extracted from murine fecal specimens using the CTAB protocol. Amplification of the 16S rRNA gene was performed using a GeneAmp® PCR System 9700 thermal cycler (Applied Biosystems, Foster City, CA, USA). Subsequently, DNA concentration was measured using a Qubit 4 fluorometer (Thermo Fisher Scientific, Waltham, MA, USA) and fragment size distribution was evaluated with a bioanalyzer (Agilent Technologies, Santa Clara, CA, USA). Library quality was further assessed with an Illumina quantification kit, and paired-end sequencing was carried out on an Illumina NovaSeq 6000 system (Illumina, San Diego, CA, USA). Raw sequencing reads were processed by demultiplexing based on barcode sequences, and paired-end reads were merged using FLASH. Low-quality sequences were filtered with FQTRIM. Amplicon Sequence Variants (ASVs) were inferred from quality-filtered reads using the DADA2 plugin in QIIME2. Taxonomic classification was performed by aligning ASVs to the SILVA database at a 0.7 confidence threshold, and relative abundances were calculated accordingly.

### 4.10. SCFA Metabolomics Detection

Standard solutions of the target analytes were prepared in deionized water for subsequent GC-MS (gas chromatography–mass spectrometry) analysis. Colon content samples were processed by vortexing and centrifugation prior to GC-MS analysis. Chromatographic separation was carried out on an Agilent HP-FFAP capillary column (30 m × 250 μm × 0.25 μm) with a 1 µL injection volume in split mode (5:1 ratio). The GC oven temperature protocol began at 50 °C, ramping to 150 °C at 50 °C/min, then to 170 °C at 10 °C/min, subsequently to 210 °C at 20 °C/min, and finally to 240 °C at 40 °C/min. Helium was employed as carrier gas maintaining a 1.0 mL/min flow rate. Mass spectrometric analysis was conducted using a SHIMADZU GC2030-QP2020 NX system (Shimadzu, Kyoto, Japan) configured with injector, transfer line, ion source, and quadrupole temperatures of 240 °C, 240 °C, 200 °C, and 150 °C, respectively. Electron impact ionization at 70 eV was utilized for data acquisition in full scan/SIM mode across *m*/*z* 33–150. Quantitative analysis of SCFAs was performed by extracting peak areas and retention times, with concentrations calculated using linear regression models generated from analyte-specific calibration curves.

### 4.11. Cell Culture

Caco-2 human colorectal adenocarcinoma cells, sourced from Wuhan Procell, were cultured under standard conditions (37 °C, 5% CO_2_) in DMEM medium containing 10% FBS and 1% penicillin–streptomycin. Upon reaching 80–90% confluency, typically every 2–3 days, cells were dissociated and subcultured at a 1:3 split ratio.

### 4.12. Cell Viability Assay

In 96-well plates, Caco-2 cells were plated at a density of 2 × 10^4^ cells per well and exposed to SMS for 24 h. The culture medium was replaced with MTT solution at 37 °C for 4 h. The medium was subsequently removed, and 150 μL of DMSO was introduced to solubilize the formazan crystals. The absorbance was quantified at 570 nm employing a microplate reader.

### 4.13. Western Blot

Colon and liver tissues, along with cultured Caco-2 cells, were lysed using RIPA buffer supplemented with 1% PMSF. The protein concentration was quantified with a BCA assay kit, followed by heat-induced denaturation. After separation by electrophoresis and transfer onto a membrane, the membrane was blocked for 2 h. The membrane was exposed to primary antibodies (1:1000) in a 4 °C overnight incubation. Following thorough washing, with the appropriate secondary antibody (1:5000), quantitative analysis of band grayscale values was performed employing ImageJ (version 1.54). For tissue samples, each lane represented one individual animal, and no samples were pooled. Densitometric analyses were based on three biologically independent animals per group (n = 3). Caco-2 experiments were repeated independently three times using separately cultured and treated cells. Each experiment included three technical replicate wells per treatment condition. Technical replicates were averaged within each experiment, and statistical analyses used the three independent experiments (n = 3). Total target proteins were normalized to β-actin, whereas phosphorylated proteins were normalized to their corresponding total proteins. The normalized control value was set to 1 for relative quantification.

### 4.14. Quantitative Real-Time PCR (qRT-PCR)

RNA from colon tissues was reverse-transcribed into cDNA after isolation with TRIzol reagent (Thermo Fisher Scientific, #15596026, USA). Subsequent qPCR was run on a QuantStudio 1 instrument (Thermo Fisher Scientific, USA) with a SYBR Green One-Step qRT-PCR kit (Beyotime Biotechnology Co., Ltd., Shanghai, China) according to the manufacturer’s guidelines. The relative expression of the target gene was determined by the 2^−ΔΔCt^ method, using β-actin for normalization. All primer sequences utilized in this assay can be found in Table 3.

### 4.15. Biochemical and Cytokine Analysis

Commercial kits were employed to assess a range of parameters: biochemical kits for hepatic lipids (HDL-C, LDL-C, TG, T-CHO) and enzymes (ALT, AST), as well as gut barrier markers (D-LA, DAO, ET, zonulin); ELISA kits for pro-inflammatory cytokines (IL-1β, IL-6, TNF-α) in tissue homogenates and supernatants; and specific assays for LPS levels in serum and liver tissues, all performed according to manufacturers’ instructions.

### 4.16. Immunofluorescence (IF) Assay

Treated Caco-2 cells underwent fixation in 4% paraformaldehyde and permeabilization with 0.5% Triton X-100 (Sigma-Aldrich, St. Louis, MO, USA), and then incubation with target-specific primary antibodies at 4 °C overnight and fluorophore-conjugated secondary antibodies, prior to nuclear labeling with DAPI. Fluorescence images were captured using an Olympus DP74 microscope (Olympus Corporation, Tokyo, Japan).

### 4.17. Statistical Analysis

Statistical analyses were performed using SPSS 25.0, and figures were prepared using GraphPad Prism 9.5.1. Data are presented as mean ± SD. Two groups were compared using two-tailed unpaired Student’s *t*-tests, whereas multiple groups were analyzed by one-way ANOVA followed by Tukey’s test. Longitudinal data were analyzed by two-way repeated-measures ANOVA with Tukey-adjusted comparisons. Normality and variance homogeneity were assessed using Shapiro–Wilk and Levene’s tests, respectively. No outliers were excluded or missing values imputed. Unless stated otherwise, n represents biological replicates; technical replicates were averaged. All tests were two-sided, with adjusted *p* < 0.05 considered significant. Microbiome analyses were performed on the Majorbio ISanger platform. Spearman correlations used paired microbiome, SCFA, and host data; unadjusted *p* values were considered exploratory. LEfSe used an LDA threshold > 3.0.

## 5. Conclusions

This study demonstrates that SMS effectively improves HFD-induced MASLD in mice. Specifically, SMS reduced hepatic accumulation of TG and T-CHO, which helped to maintain normal liver physiological function. Notably, these effects occurred alongside changes in gut microbial composition and SCFA concentrations in colonic contents, improved colon histology and tight-junction-associated measures, lowered circulating LPS, and reduced TLR4/NF-κB-associated signaling. The Caco-2 experiments showed attenuation of LPS-induced inflammatory responses and were not used to assess epithelial permeability. In summary, our study collectively indicates that SMS exerts its therapeutic effects through a multi-targeted, synergistic mechanism. This provides a conceptual framework for developing Tibetan medicine-based intervention strategies for MASLD targeting the gut–liver axis.

## Figures and Tables

**Table 2:** Chromatographic conditions for HPLC analysis.

Time (min)	0.1% Phosphoric Acid Water (A)	Acetonitrile (B)	Detection Wavelength	Flow Rate	Injection Volume	Column Temperature
0	98%	2%	220 nm	1 mL/min	1 µL	20 °C
30	98%	2%				

**Table 3:** qRT-PCR primer sequences.

Gene	Forward Primer	Reverse Primer
*ZO-1*	TCAAAGTCTGCAGAGACAATAGCAT	GCCAAGTTTTAGGGTCACAGTGT
*Occludin*	CGACAGTCCGATGGCCTACT	TACCGAGGCTGCCTGAAGTC
*Claudin*	ATGTCCTGCGTTTCGCAAAG	GCCAATGGTGGACACAAAGAT
*β-actin*	CCTGTGGCATCCATGAAACTAC	CCAGGGCAGTAATCTCCTTCTG

## Data Availability

The original contributions presented in this study are included in the article and Supplementary Materials. Further inquiries can be directed to the corresponding authors.

## Supplementary Materials

The following supporting information can be downloaded at https://www.mdpi.com/article/10.3390/ijms27167368/s1.

## Abbreviations

The following abbreviations are used in this manuscript:

SMS	*Sophora moorcroftiana* seed ethanol extract
MASLD	metabolic dysfunction-associated steatotic liver disease
MASH	metabolic dysfunction-associated steatohepatitis
HFD	high-fat diet
LPS	lipopolysaccharide
TLR4	Toll-like receptor 4
MyD88	myeloid differentiation primary response 88
ZO-1	zonula occludens-1
DAO	diamine oxidase
D-LA	D-lactic acid
IHC	immunohistochemistry
qRT-PCR	quantitative real-time polymerase chain reaction
ASV	amplicon sequence variant
LEfSe	linear discriminant analysis effect size
PICRUSt	phylogenetic investigation of communities by reconstruction of unobserved states
OPLS-DA	orthogonal partial least squares discriminant analysis
CON	normal-diet control group
L-SMS	low-dose SMS group
M-SMS	medium-dose SMS group
H-SMS	high-dose SMS group
SCFAs	short-chain fatty acids
ALT	alanine aminotransferase
AST	aspartate aminotransferase
TGs	triglycerides
T-CHO	total cholesterol
LDL-C	low-density lipoprotein cholesterol
HDL-C	high-density lipoprotein cholesterol
PCA	principal component analysis
PCoA	principal coordinate analysis
NMDS	non-metric multidimensional scaling
KEGG	Kyoto Encyclopedia of Genes and Genomes
GC-MS	gas chromatography–mass spectrometry
