# Supplementary material for: Sophora moorcroftiana Seeds Ethanol Extract Against Metabolic Dysfunction-Associated Steatotic Liver Disease in Mice by Modulating Gut Microbiota Dysbiosis, SCFAs, and Related Inflammation
Source: Int J Mol Sci. 2026 Aug 18;27(16):7368. doi: 10.3390/ijms27167368 (PMC13513855; doi:10.3390/ijms27167368)
Supplement: Supplementary file 1 [file ijms-27-07368-s001.zip › ijms-4408392-supplementary.pdf]

Supporting Information for

ORIGINAL ARTICLE

Sophora moorcroftiana Seeds Ethanol Extract Against Metabolic Dysfunction-Associated Steatotic Liver Disease in Mice by Modulating Gut Microbiota Dysbiosis, SCFAs, and Related Inflammation

Xiaotong Chu [1], Shuang Zhang [1] , Mingxue Cui [1] , Xiaojing Sun [1], Xiao Chen [1], Liying Gao [1], Ruiying Yuan [2], Sicen Wang [2,3], Shan Huang [1,*], Bin Li [1,2,*]

[1] Department of Pharmaceutical Engineering and Pharmacy, School of Chemical Engineering, Qingdao University of Science and Technology, Qingdao 266042, China; 17205228560@163.com (X.C.); zhangshuang9797@163.com (S.Z.); cuimx726@163.com (M.C.); s10240513@163.com (X.S.); 19819197685@163.com (X.C.); ggaoliying@163.com (L.G.)

[2] Department of Pharmacy, College of Medicine, Tibet University, Lhasa 850001, China; 1yuan2yuan@163.com (R.Y.); wangsc@mail.xjtu.edu.cn (S.W.)

[3] College of Pharmacy, Xi’an Jiaotong University, Xi’an 710061, China

* Correspondence: huangshan@qust.edu.cn (S.H.); leebin09@qust.edu.cn (B.L.)

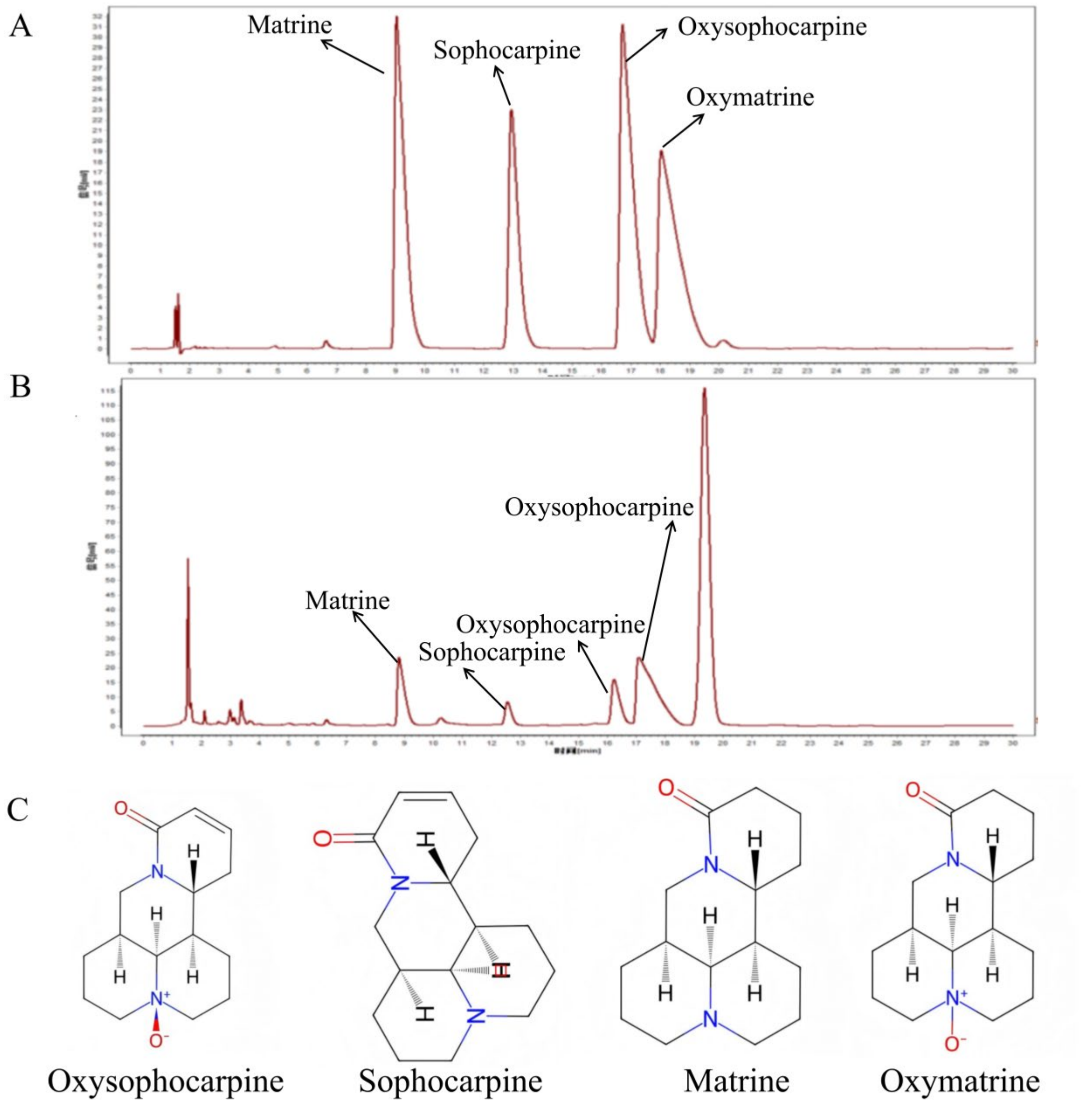

Fig.S1. Representative components in SMS were analyzed by HPLC.

HPLC chromatograms of four reference standards (A) and SMS sample (B). (C) Chemical structures of oxysophocarpine, sophocarpine, matrine, and oxymatrine.

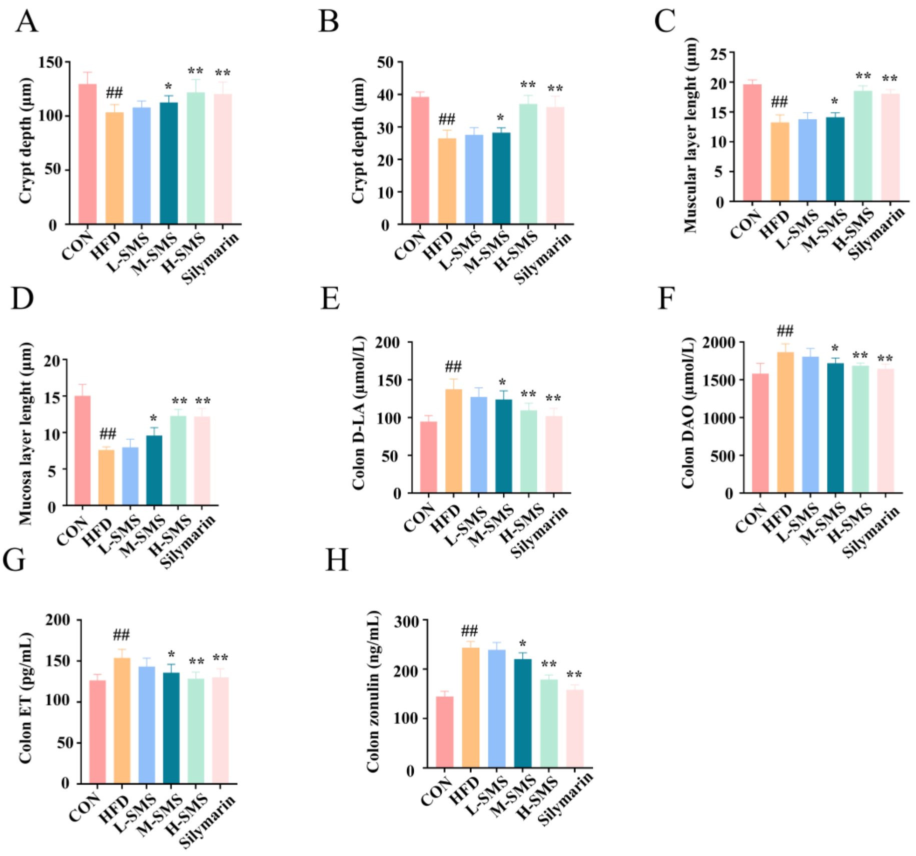

Fig.S2. Effect of SMS on colon morphology and intestinal permeability in HFD-induced MASLD mice Morphometric analysis was conducted to evaluate colon (A) villus length, (B) crypt depth, (C) muscular layer length, and (D) mucosa layer length. (E-H) The concentrations of DAO, ET, D-LA, and zonulin, in mouse colon tissue. Data are presented as mean ± SD (n = 8). $^{\#}P < 0.05$, $^{\#\#}P < 0.01$ compared to the CON group, $^{*}P < 0.05$, $^{**}P < 0.01$ compared to the HFD group.

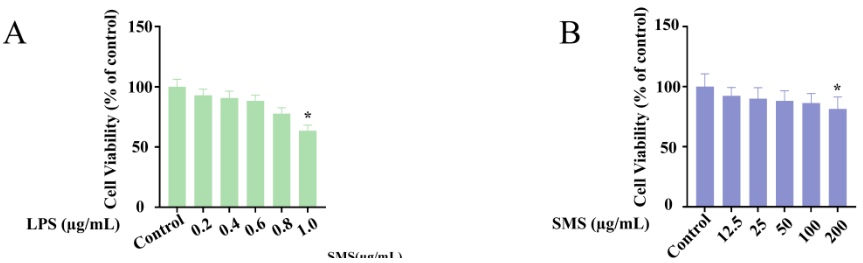

Fig.S3. Effect of SMS on LPS-induced damage in Caco-2 cells.
(A) Caco-2 cells were treated with different concentrations of LPS (0.2 μg/mL to 1.0 μg/mL) for 24 h.
(B) The effects of various concentrations of SMS on Caco-2 cells viability for 24 h were evaluated.
Data are mean ± SD from three independent experiments (n = 3); three technical replicate wells per treatment condition were averaged within each experiment. $^{\#}P < 0.05$, $^{\#\#}P < 0.01$ compared to the CON group, $^{*}P < 0.05$, $^{**}P < 0.01$ compared to the HFD group.

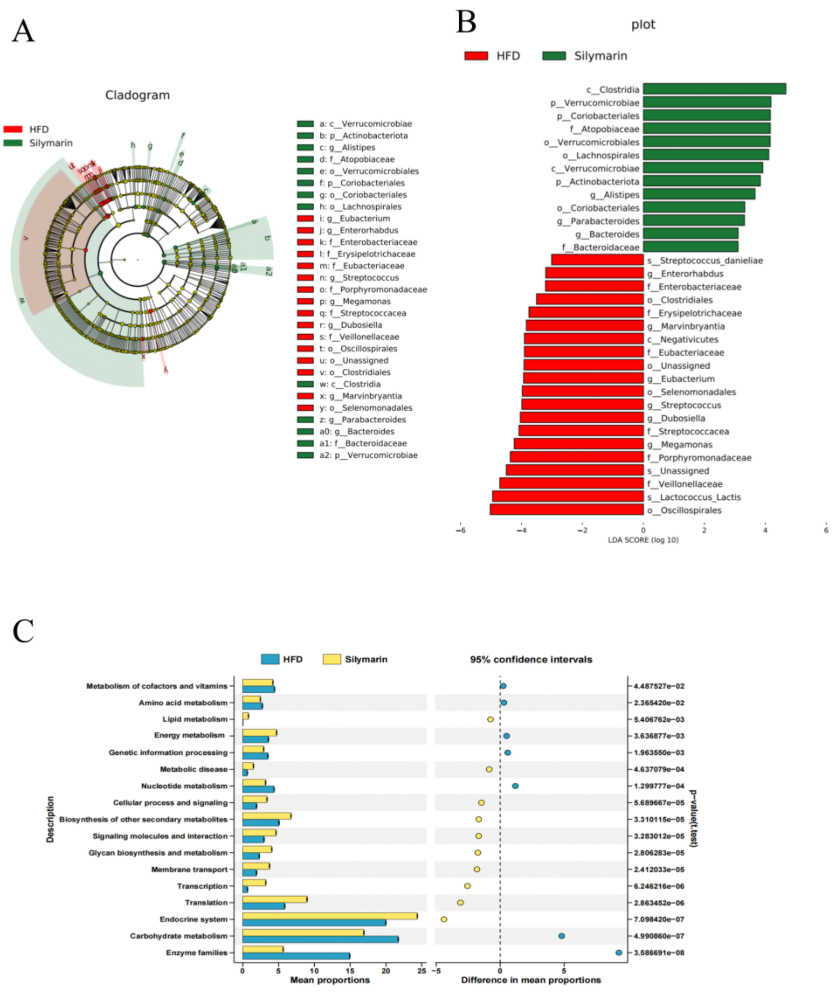

Fig.S4. Effect of Silymarin on LEfSe analysis and metabolic functions of gut microbiota. (A) LEfSe analysis and (B) LDA score for characteristic bacterial taxa. (C) Significant functional abundance differences between HFD group and Silymarin group in KEGG pathway.

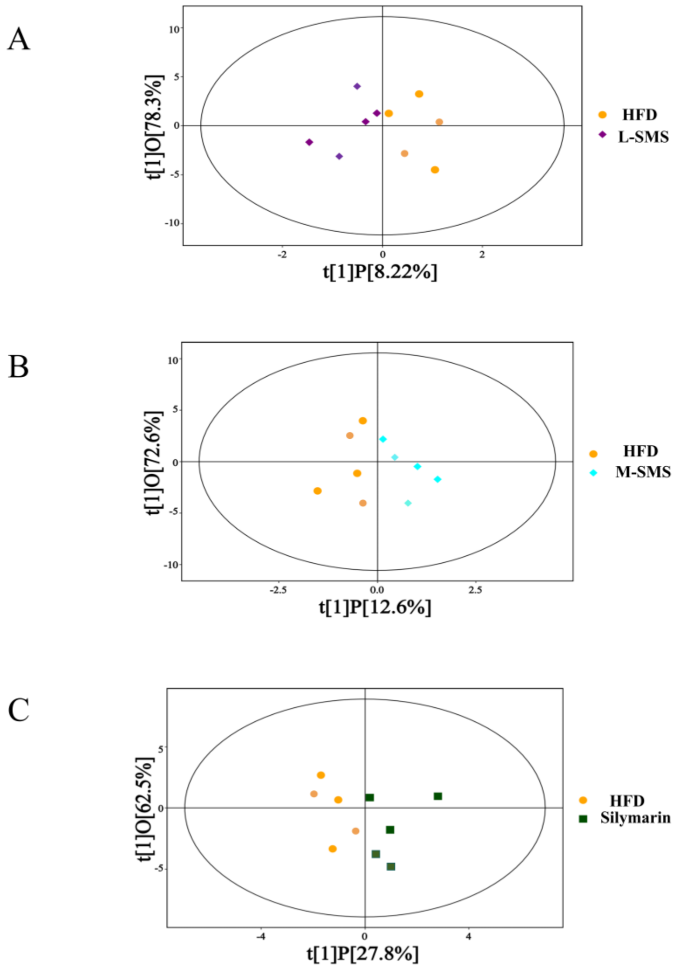

Fig.S5. Effect of SMS on the metabolic disorder of SCFAs in HFD-induced MASLD mice.

(A-C) The OPLS-DA score plot of the HFD vs. L-SMS groups, the HFD group vs. M-SMS groups and the HFD group vs. Silymarin groups (n = 5).
